# On Arsenical Solution in Hooping Cough

**Published:** 1811-02

**Authors:** 


*To the Editors of the Medical and Physical Journal.*

GENTLEMEN,

IN looking over the three volumes of "Medical Histories and Reflections," by Dr. Ferriar, *second edition*, lately published, I found in the third volume under the head of "Hooping Cough," page 221, the following observations.—

"I believe that the only remedy, which promises to shorten the disease effectually, is the solution of white arsenic. I have employed this medicine in several cases of infirmary patients, with tolerable success; and I have occasionally given it in private practice, with so much advantage, that I think it deserving of farther trial. The dose with which I

 generally generally begin is *one drop*, daily, for an infant; and for children under seven, *two drops*, repeated according to the state of the symptoms. It requires some caution, to avoid the accumulated action of this medicine. The exhibition of the solution should be suspended occasionally, for a day or more, and the bowels should be gently opened by means of a little calomel."

As there are no marks by which one can judge, whether the above extract is given as the result of Dr. Ferriar's experience, *since* the publication of the former edition of these volumes, or whether it now remains as it was in the first edition, I shall be much obliged to any of your correspondents who may be in possession of that edition for information on the subject. If the above observation be now for the first time published, the reputation of the author will undoubtedly induce other practitioners to make a trial of the remedy proposed; but if, as I suspect, this passage is to be found in the former edition, there cannot be reasonably entertained much expectation of advantage from the use of arsenic in the hooping-cough;—for it amounts to this: Dr. Ferriar's third volume was originally published ten years ago, during which time he must have had repeated opportunities of exhibiting this medicine in the hooping-cough; of course, if after ten years additional experience, in so common a complaint, he still speaks *doubtingly*, we may fairly infer, that the solution of arsenic is not to be depended upon, and consequently, as Dr. Ferriar admits that it is a hazardous medicine, it would be wise to discard it.

I observe likewise in Dr. Ferriar's second volume, a recommendation of an infusion of digitalis to be used as a lotion, in cancerous cases: Has this been tried for cancer? It does not appear that Dr. F. used it as a remedy for cancer, but he says, that the simple infusion of digitalis gave relief "in a very painful and ulcerated herpetic affection of the face, which was irritated by the most simple applications, and which would not bear the mildest preparations of lead;" and hence he is led to suppose that it might relieve cancerous ulcers.

Information from any of your Correspondents on these points, will much oblige

Your constant Reader,

M.

*December* 12, 1810.

